# The effectiveness of older insecticide-treated bed nets (ITNs) to prevent malaria infection in an area of moderate pyrethroid resistance: results from a cohort study in Malawi

**DOI:** 10.1186/s12936-020-3106-2

**Published:** 2020-01-15

**Authors:** Monica P. Shah, Laura C. Steinhardt, Dyson Mwandama, Themba Mzilahowa, John E. Gimnig, Andy Bauleni, Jacklyn Wong, Ryan Wiegand, Don P. Mathanga, Kim A. Lindblade

**Affiliations:** 1 Malaria Branch, Division of Parasitic Diseases and Malaria, Center for Global Health, U.S. Centers for Disease Control and Prevention, Atlanta, GA USA; 2 College of Medicine, Malaria Alert Centre, Blantyre, Malawi; 3 Entomology Branch, Division of Parasitic Diseases and Malaria, Center for Global Health, U.S. Centers for Disease Control and Prevention, Atlanta, GA USA

**Keywords:** Malaria, Insecticide-treated bed nets, Prevention, Vector control, Insecticide resistance, Holes

## Abstract

**Background:**

A previous cohort study in Malawi showed that users of new insecticide-treated bed nets (ITNs) were significantly protected against malaria compared to non-users, despite moderate levels of pyrethroid resistance among the primary mosquito vectors. The present study investigated whether ITNs that were 1–2 years old continued to protect users in the same area with moderate pyrethroid resistance.

**Methods:**

One year following a baseline cross-sectional malaria parasitaemia prevalence survey and universal distribution of deltamethrin ITNs (May 2012), a fixed cohort of 1223 children aged 6–59 months was enrolled (April 2013). Children were tested for parasitaemia at monthly scheduled visits and at unscheduled sick visits from May to December 2013 using rapid diagnostic tests. ITN use the prior night and the condition of ITNs (based on presence of holes) was assessed by caregiver self-report. The incidence rate ratio (RR) comparing malaria infection among users and non-users of ITNs was modelled using generalized estimating equations adjusting for potential confounders and accounting for repeated measures on each child. The protective efficacy (PE) of ITN use was calculated as 1 − RR.

**Results:**

In this cohort, self-reported ITN use remained consistently high (> 95%) over the study period. Although users of ITNs were slightly more protected compared to non-users of ITNs, the difference in incidence of infection was not statistically significant (RR 0.83, 95% confidence interval [CI] 0.54–1.27). Among ITN users, malaria incidence was significantly lower in users of ITNs with no holes (of any size) compared to users of ITNs with ≥ 1 hole (RR 0.82, 95% CI 0.69–0.98).

**Conclusions:**

There was no significant PE of using 1–2 year-old ITNs on the incidence of malaria in children in an area of moderate pyrethroid resistance, but among ITN users, the authors found increased protection by ITNs with no holes compared to ITNs with holes. Given the moderate levels of pyrethroid resistance in the primary malaria vector and recent evidence of added benefits of ITNs with synergists or non-pyrethroid insecticides, next-generation ITNs may be a useful strategy to address pyrethroid resistance and should be further explored in Malawi.

## Background

Insecticide-treated bed nets (ITNs) have contributed substantially to declines in malaria morbidity and all-cause mortality across sub-Saharan Africa [1, 2]. ITNs prevent mosquito blood feeding by physically inhibiting human-mosquito contact and by chemically deterring, irritating, and killing mosquitoes. The physical barrier of the ITN prevents mosquito entry, while the pyrethroid treatment induces mosquito excito-repellency and causes paralysis leading to mosquito death. At high levels of coverage, ITNs reduce the overall density and life-span of mosquito populations, resulting in community-wide malaria protection both for those sleeping under ITNs and for neighbouring non-users of ITNs [3].

The emergence of mosquito resistance to pyrethroids has led to concerns about the continued effectiveness of ITNs. Resistance can occur due to upregulation of metabolic enzymes, which promote faster clearance of the insecticide, and/or mutations in target site genes, such as the knockdown resistance gene (*kdr*), which limit the binding of the insecticide to initiate paralysis. Either of these mechanisms leads to reduced deterrence and mortality, which could compromise the mass killing effect of ITNs. However, ITNs may continue to provide partial protection through other mechanisms. To date, entomologic evidence from a meta-analysis of laboratory and field studies has shown that the effectiveness of ITNs was not significantly reduced due to insecticide resistance alone [4]. Similarly, epidemiologic evidence from studies in Benin [5], Côte d’Ivoire [6], Malawi [7], Kenya [8], and a multi-country study [9] have shown that ITN use remains protective against malaria infection even in areas with moderate-to-high levels of pyrethroid resistance. However, it is unclear whether ITNs will continue to be effective in areas with pyrethroid resistance as the nets develop holes or tears over time and the insecticide content on the net declines.

Entomologic studies have shown that the development of holes in nets combined with pyrethroid resistance leads to increased mosquito blood feeding. Experimental hut trials conducted in an area of high pyrethroid resistance in Benin found that blood-feeding rates in *Anopheles gambiae* were almost three times greater in ITNs with 80 holes (32%) compared to ITNs with 6 holes (12%) [10]. In another experimental hut study conducted in two sites in Benin with different levels of pyrethroid resistance, among ITNs that were cut to simulate holed nets, blood feeding was substantially reduced in the site where mosquitoes were susceptible, while no impact on blood feeding was observed in the site with high levels of pyrethroid resistance [11]. In an area of high pyrethroid resistance in western Kenya, a significantly higher number of mosquitoes was observed resting inside ITNs with larger hole areas compared to ITNs with no holes (risk ratio 1.9; 95% confidence interval [CI] 1.2–3.1) [12]. Although these findings are consistent with reduced deterrence due to pyrethroid resistance, studies examining the outcome of mosquito mortality have been mixed; similar rates of mosquito mortality were observed in experimental huts using ITNs with and without holes in a setting of pyrethroid-resistant *Culex quinquefasciatus* and *An. gambiae* sensu lato (s.l.) [13], while another study of equivalently-holed ITNs found significantly reduced mortality in a setting of high *kdr* resistance (19%) compared to a setting of *kdr* susceptibility (98%) [11].

The development of holes in ITNs in settings of pyrethroid resistance may increase blood feeding and potentially reduce mosquito mortality, but few studies have examined whether this compromises the operational effectiveness of ITNs. The use of older nets and nets with holes compared to no use of nets was associated with reduced odds of malaria in cross-sectional surveys of children in Benin [14], Equatorial Guinea [15], and Malawi [15]; however, the protective effect decreased with increasing deterioration of the nets. In Malawi, which has moderate levels of pyrethroid resistance [7], repeat analysis of cross-sectional survey data for 3 years following a national distribution campaign found that ITN use was associated with reduced prevalence of malaria in children aged 5–15 years (school-aged), but associations were null among other age groups [16]. A health facility-based case–control study conducted in children aged 6–59 months found no significant personal protective effect of ITNs approximately 1 year after ITNs were distributed in an area of moderate pyrethroid resistance in Malawi [17]. Given the potential for selection bias introduced by health facility-based controls [18] and limitations of cross-sectional surveys, results from prospective studies are needed to understand if protection by ITNs becomes compromised once holes develop in settings of pyrethroid resistance.

In 2012–2013, a cohort study of children aged 6–59 months was carried out in an area of moderate pyrethroid resistance in Malawi to determine the protective efficacy (PE) of newly distributed ITNs on incidence of malaria parasitaemia. Over the 1-year study period, ITN use was associated with a significant PE of 30% (rate ratio [RR] 0.7; 95% CI 0.5–0.8) compared to no use of ITNs [7]. Although this study demonstrated that ITNs were still effective in settings of moderate pyrethroid resistance, the ability of ITNs to continue to prevent malaria infection once the physical condition of nets degraded and insecticide content declined over time remained uncertain. The objective of the present study was to evaluate the PE of ITNs 1–2 years after use in a setting of moderate pyrethroid resistance.

## Methods

### Study area and population

This study was a follow-up to a cohort study (first cohort) conducted by Lindblade et al. [7] to determine the PE of ITNs on incidence of malaria parasitaemia in an area of moderate pyrethroid resistance in Malawi. The entire population of Malawi is at risk of malaria infection by *Plasmodium falciparum* year-round, with heightened transmission during the rainy season between December and March. Universal coverage of ITNs has been a major component of the malaria control strategy and, between 2010 and 2014, the proportion of households owning at least one ITN increased from 58 to 70% following several universal distribution campaigns [19, 20].

**Fig. 1:**
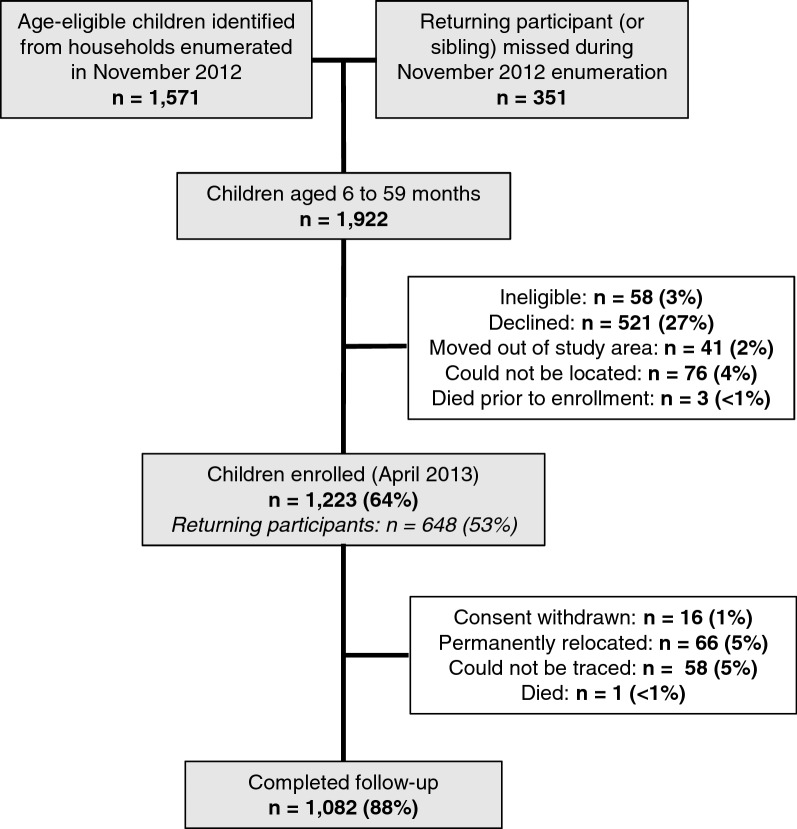
Study profile of second cohort, Liwonde, Malawi 2013

The study area included the same six villages in the Traditional Authority of Sitola, Machinga District, Malawi, where the first cohort study was conducted. All households in the villages were mapped and enumerated in February–March 2012, just preceding the enrollment of the first cohort in March 2012, which was followed until March 2013. A second census was carried out in November 2012, and all children 6–59 months old as of April 1, 2013 were invited to participate in the present study (Fig. 1). The new, fixed cohort of children (second cohort) was enrolled in April 2013 and followed from May to December 2013.

Entomologic surveillance occurred every fortnight in 10 clusters of the study area during the first cohort and ended at the start of the second cohort. Just prior to the enrollment of the second cohort (March 2013), mortality of *Anopheles funestus* measured by World Health Organization (WHO) resistance assays ranged from 8 to 31% following exposure to deltamethrin 0.05% and from 13 to 67% following exposure to permethrin 0.75%, respectively. Mortality was slightly higher in *An. gambiae* s.l., at 53% as measured from a single village in Machinga district exposed to deltamethrin 0.05% and ranging from 6 to 91% in four villages in Machinga and neighbouring Balaka district exposed to permethrin 0.75%. From mosquitoes collected during the first cohort, the primary mechanism of resistance was found to be metabolic with no evidence of target site mutation in *kdr*.

### Sample size determination

The sample size for the second cohort was determined based on the same assumptions as the first cohort [7]. Using a generalized estimating equations (GEE) approach [21], assuming a Poisson distribution with exchangeable correlation structure for repeated measurements on children and an incidence rate of malaria parasitaemia among children not using ITNs of 1.0 episode per person-year (assuming 30% of children would not use ITNs), the sample size required for 70% power to detect a 30% reduction in incidence of malaria parasitaemia among ITN users at a two-sided alpha equal to 5% was 981 children. After accounting for a 5% refusal rate, 15% loss-to-follow-up over 9 months, and a mortality rate of 1.5% per year, enrollment was planned for 1335 children.

### Enrollment, monthly visits and sick visits

Similar procedures for enrollment, monthly visits and unscheduled sick visits were followed for the first [7] and second cohorts. Children were eligible to participate if they were 6–59 months old, not taking daily cotrimoxazole for human immunodeficiency virus infection or exposure, weighed 5 kg or more, and planned to stay in the study area for at least 1 month. Participants of the first cohort study could enroll in the second cohort if they met the eligibility criteria. Enrollment and monthly visits took place at central locations within the communities, and no incentives or transportation reimbursement were provided to attend scheduled monthly visits. Malaria parasitaemia was measured at scheduled monthly visits and at unscheduled sick visits if the child fell ill in between monthly visits. Caregivers were encouraged to bring their children for examination by a study clinician at Machinga District Hospital, one of the only health care providers in the area, for any illnesses occurring between scheduled visits. Caregivers were reimbursed for transportation to the study clinic for up to one unscheduled sick visit per month.

At enrollment, monthly visits and unscheduled sick visits, caregivers were interviewed about their child’s history of illness over the past 2 weeks, use of anti-malarials, and use of ITNs the night prior to the visit. Caregivers were also asked about the condition of the child’s ITN, including age and the presence of holes of any size, holes between fist and head-sized (large holes, 10–25 cm), or holes that were head-sized or larger (extra-large holes, > 25 cm) [22]. The child’s axillary temperature was measured using a thermometer and a blood sample was collected by finger stick for malaria testing and haemoglobin measurement (g/dL). Malaria testing was performed using histidine-rich protein 2 (HRP-2) *P. falciparum* rapid diagnostic test (RDT; SD Bioline Malaria Ag Pf^®^ ref. 05FK53, Kyonggi-do, Republic of Korea). Haemoglobin was measured using a Hemocue^®^ (Angelholm, Sweden) portable haemoglobinometer. Children with positive RDT results were treated with an appropriate anti-malarial as per national guidelines.

At enrollment only, all children were cleared of parasites with a full, weight-appropriate treatment course of dispersible artemether-lumefantrine (Coartem^®^-D, Novartis, Basel, Switzerland). Dried blood spots were also collected on filter paper for molecular testing by polymerase chain reaction (PCR) for *P. falciparum, Plasmodium vivax, Plasmodium ovale,* or *Plasmodium malariae* to determine malaria prevalence at enrollment. Anthropometric measures of height and weight were taken.

Study staff made three attempts within 1 week to complete a follow-up visit for children who missed their scheduled monthly visit. Children who missed three or more consecutive scheduled visits were withdrawn from the study.

### ITN ownership and use

Study staff distributed Permanet 2.0 long-lasting ITNs (Vestergaard, Lausanne, Switzerland), treated with deltamethrin, in the study area in May 2012, just after enrollment of the first cohort. One ITN was distributed per two individuals in each household, and an additional ITN was provided for households with an odd number of residents. Outside of the study area, the National Malaria Control Programme of Malawi conducted a national ITN distribution campaign with Olyset Nets (Sumitomo Chemical Co., Japan) in July 2012 using the same allocation scheme. No additional ITN distributions were conducted in the study area after the study distribution; however, during enrollment of the second cohort, caregivers of participants were provided with an ITN by the study if the child’s ITN was reported to be lost, stolen, or damaged. All bed nets were considered ITNs following the study distribution in May 2012.

At each study visit, caregivers were asked about their child’s ITN use the night before the visit, during the 2-week period before the visit, and about “typical use” during the current season. The reliability of caregiver-reported ITN use was assessed in two cross-sectional surveys employing home visits during the first and second cohort studies; the proportion of positive agreement between caregiver-reported and actual use was high (> 93%), while the proportion of agreement in negative responses was low (< 29%) [23].

### Data management and analysis

To explore the effect of holes on the effectiveness of ITNs, the caregiver-reported condition of ITNs used by study participants was categorized as i) use of an ITN with no holes, use of an ITN with a small or medium hole (no large or extra-large holes), use of an ITN with a large or extra-large hole, and ii) use of an ITN with any holes *vs.* use of an ITN with no holes. Children with haemoglobin levels < 11 grams per deciliter were considered anaemic. Age- and sex-specific WHO growth reference charts [24] were used to classify stunting (height-for-age) or wasting (weight-for-age) in children with Z-scores that were ≤ 2 standard deviations below the median at enrollment.

Several variables were considered as potential confounders in this analysis based on previous associations documented in the literature. Age was dichotomized as 6 to < 12 months and 12 months or older. A dummy variable to indicate whether the child was a returning member of the first cohort was included. To control for the geographic heterogeneity of malaria transmission, individual risk of malaria infection was estimated using inverse distance-weighted (IDW) malaria prevalence at enrollment within 1 km of each child. Study visits were categorized as occurring during high (April–June 2013) or low (July–December 2013) malaria season. To account for the community protection conferred by widespread use of ITNs, the number of ITNs within a 300-m radius [3] as enumerated during the November 2012 census was included. Altitude of the household was measured at enrollment. Household wealth was differentiated by principal components analysis of household assets [25]. The following household assets were included: electricity, paraffin lamp, battery lamp, radio, television, cell phone, mattress, sofa set, table and chairs, refrigerator, bicycle, motorcycle, car, source of water, type of toilet, type of floor material, type of roof material, type of wall material and number of sleeping rooms. Educational attainment of the primary caretaker was dichotomized into completion of primary school (or greater) versus no completion of primary school. All continuous variables were divided into terciles and analysed categorically.

Monthly and unscheduled sick visits were combined for analysis and person-time at risk accumulated from enrollment through the final study encounter (either a monthly visit or unscheduled sick visit) was calculated for each child. Children who were withdrawn from the study were censored at their last encounter date. Person-time at risk was adjusted for malaria treatment and diagnostic characteristics. The half-life of lumefantrine (10.5 days) [26] was subtracted from the person-time at risk following the baseline parasite clearance dose. In addition, the HRP-2 parasite antigen detected by RDTs can persist in peripheral blood after an infection has been cleared, potentially resulting in a false positive test result. Based on data from previous studies [27, 28], the at-risk time for participants with consecutive positive RDTs was removed such that a second positive result occurring < 17 days from a prior positive visit was considered as a false positive, while consecutive positive results ≥ 17 days apart were considered as new infections and contributed towards person-time at risk.

Malaria incidence was modelled using Poisson regression with log-transformed person-time as an offset in GEE [29] using SAS version 9.4 (SAS Institute, Inc., Cary, NC, USA) and the GENMOD procedure. Analysis using GEE was selected because estimates are robust and amenable to misspecification of correlation structure for a large number of clusters [30]. Fixed effects were estimated by least squares means and standard error estimates for model parameters were adjusted for repeated measures by considering an independent working correlation structure. An independent correlation structure was selected because parameter estimates using other correlation structures (exchangeable and unstructured) were unstable. The full model included the main predictor of interest, sleeping under an ITN, which was treated as time-varying, and all potential confounders based on reported associations in literature, including both time-variant and time-invariant variables. Collinearity was assessed from an information matrix for variables with condition indices greater than 30 with two or more variance decomposition proportions greater than 0.5 [31]. Potential confounders were evaluated using the 10% change in estimate approach, in which the change in effect measures for ITN use were compared for all possible subsets (combinations) of covariates to identify reduced models that sufficiently control for confounding [32, 33]. The final model was selected among the reduced models based on precision (smaller standard error) of the effect estimate for ITN use and model fit statistics (lower Akaike Information Criterion). Adjusted incidence per person-year (iPPY) was estimated for each variable of interest by assigning mean values for other dependent variables. Rate ratios (RR) and 95% CIs were estimated from models accounting for correlated data (univariate and multivariate analyses) and adjusted for confounders (multivariate analysis). PE was calculated as 100%*(1 − R_1_/R_0_) where R_1_ is the rate of malaria infection among ITN users and R_0_ is the rate among non-ITN users. p-values < 0.05 were considered statistically significant.

## Results

### Coverage and use of insecticide-treated bed nets in the study area

As measured in the censuses conducted in the study area in households with and without enrolled children, the proportion of households with at least one ITN per two residents (universal coverage) doubled following the ITN distribution campaigns from 23% (95% CI 22–25%) in February 2012 to 56% (95% CI 54–58%) in November 2012. ITN use among all residents increased from 58% (95% CI 57–59%) to 70% (95% CI 69–71%) in February 2012 and November 2012, respectively.

### Cohort profile

A total of 1922 age-eligible children from two sources: 1571 children with birth dates that would put them between 6 and 59 months of age as of April 2013 were identified from 2657 households that were mapped and enumerated in November 2012 (second census) and an additional 351 children were identified at the time of enrollment. These 351 children were not enumerated during the second census, but were either age-eligible members of the first cohort or a sibling of a first cohort participant. A total of 1223 children were enrolled in April 2013 from 980 households and, among these, 648 (53%) children were returning participants of the first cohort study. Children were not enrolled due to the following reasons: 58 (3%) did not meet additional eligibility criteria, 521 (27%) declined to participate (mainly due to study fatigue), 41 (2%) had moved out of the study area, 76 (4%) could not be located, and 3 (< 1%) died prior to enrollment (Fig. 1). Characteristics of enrolled and unenrolled children were compared among the 1571 children identified from the second census; participating and non-participating children did not differ significantly by gender (Chi squared test p-value = 0.489) or age (median ages 27 and 28 months, respectively; Wilcoxon signed-rank test p-value = 0.310). However, enrolled children were significantly more likely to sleep under an ITN (84%) compared to children who were not enrolled (75%; Chi square test p-value < 0.001).

Enrolled participants made 10,566 encounters with the study between April and December 2013. The median follow-up time was 0.66 years (IQR 0.65–0.67) per child, and 1082 (89%) children completed 8 months of follow-up. Reasons for incomplete follow-up included: caretaker withdrawal of consent (n = 16, 1%), permanent relocation outside the study area (n = 66, 5%), inability to be traced (n = 58, 5%) or death (n = 1, 0%) (Fig. 1). Monthly visit attendance ranged from 91% (May 2013) to 84% (December 2013). More than half (58%) of children made at least one unscheduled sick visit and, among those children, the median number of unscheduled visits was 2 (IQR 1–4).

### Baseline characteristics

At baseline of the second cohort, a quarter (25%, 95% CI 22–27%) of children were infected with *P. falciparum* by PCR, which was significantly lower compared to the baseline malaria prevalence of the first cohort (37%, 95% CI 34–40%, [7]). Caregivers reported that most (98%) children slept under an ITN the night prior to the survey. At enrollment, 34% of participants slept under an ITN with at least one hole of any size, 14% slept under an ITN with ≥ 1 large sized hole, and 3% slept under ITNs with ≥ 1 extra-large sized hole (3%) (Table 1). Most ITNs under which children slept were approximately 1 year old (84%) and reported to be from the study ITN distribution (73%). The remaining ITNs were obtained from the National Malaria Control Programme’s universal coverage campaign in July 2012 (9%), hospital/health centres (14%), or from shops or markets (3%). At subsequent monthly visits, a majority of ITNs (> 83%) were obtained from the study or national distributions and fewer than 5% each month were reported to be obtained from shops or markets.

**Table 1:** Descriptive characteristics of the study cohort, Liwonde, Malawi April—December 2013

Characteristic	Result
Number of children enrolled	1223
Median age at baseline in months (IQR)	32 (20,45)
Number of children who completed 8 months of follow-up, n (%)	1082 (89)
Total person-years at risk over study period	640
Female, n (%)	598 (49)
*Plasmodium* infection at baseline by polymerase chain reaction, n/N (%)	300/1222 (25)
Median inverse distance-weighted malaria prevalence < 1 km at baseline (IQR)	24% (14%, 33%)
Median altitude of household, m (IQR)	497 (486, 520)
Anemic at baseline (haemoglobin < 11 g/dl), n/N (%)	546/1212 (45)
Stunting at baseline, n/N (%)	468/1174 (40)
Wasting at baseline, n/N (%)	180/1217 (15)
Caregiver completed primary school, n/N (%)	258/1217 (21)
ITN used at baseline, n/N (%)	1186/1216 (98)
ITN holes at baseline, n/N (%)	
≥1 hole of any size	415/1183 (35)
≥1 hole between fist and head-sized	168/1175 (14)
≥1 hole head-sized or larger	32/1176 (3)
Median number of ITNs within 300 m at baseline^a^ (IQR)	35 (22,79)
Total malaria infections over the study period^b^	825
Median malaria infections per person-year (IQR)	0 (0, 1.8)

*IQR* interquartile range, *ITN* insecticide-treated bed net

^a^ITN density determined from a census conducted in the study area in November 2012 (6 months prior to enrollment)

^b^As measured by malaria rapid diagnostic tests

### Protective effectiveness of insecticide-treated bed nets

**Fig. 2:**
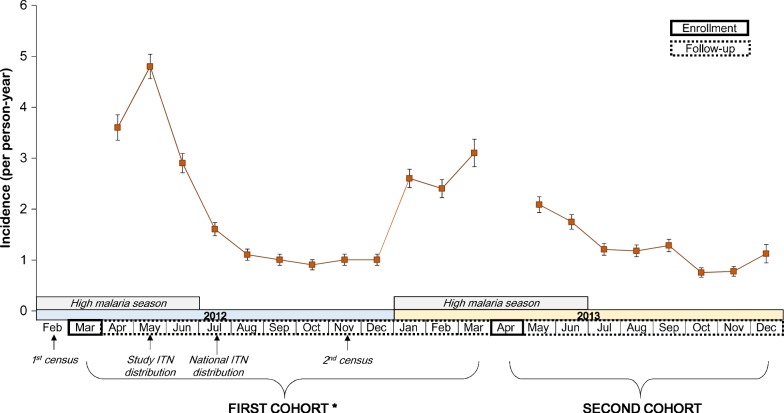
Monthly incidence of malaria infection and timeline of study activities in first and second cohorts, Liwonde, Malawi. Error bars indicate standard error of rate. * Published data from Lindblade et al. [7]

Over the 8-month study period, cohort participants experienced 825 total malaria infections and contributed 640 person-years at risk (PYAR) for a crude incidence of 1.3 iPPY and median iPPY of 0 (interquartile range 0–1.8) (Table 1). Malaria incidence was highest in May and June 2013 following the rainy season, but remained markedly lower in comparison to the same period during the first cohort (Fig. 2). By the end of the study period, 64% of children remained malaria-free while 19% were infected at least twice.

ITN use the night prior to the survey was recorded for 620 PYAR (97%) and no ITN use was recorded for 19 PYAR (3%) (Table 2, rows 5–6). The proportion of children at each monthly visit who slept under an ITN remained consistently high (between 96.4 and 98.5%) from May to December 2013.

**Table 2:** Predictors of malaria incidence in a fixed cohort of 1,223 children, Liwonde, Malawi April—December 2013

Characteristic	Malaria infections, n	PYs at risk	Univariate			Multivariate		
			Observed iPPY (95% CI)	Rate ratio (95% CI)	p-value	Adjusted iPPY (95% CI)	Rate ratio (95% CI)	p-value
Age (months)								
6–11	18	19	0.90 (0.52–1.57)	0.70 (0.40–1.22)	0.211	0.63 (0.35–1.12)	0.51 (0.3–0.89)	0.017
12+	805	620	1.29 (1.16–1.44)	Reference	–	1.22 (0.94–1.59)	Reference	–
Sex								
Female	396	317	1.24 (1.06–1.45)	0.95 (0.76–1.18)	0.626			
Male	429	322	1.31 (1.13–1.52)	Reference	–			
ITN use								
Yes	798	620	1.27 (1.14–1.42)	0.94 (0.60–1.49)	0.801	0.80 (0.59–1.08)	0.83 (0.54–1.27)	0.389
No	25	19	1.35 (0.86–2.13)	Reference	–	0.96 (0.58–1.6)	Reference	–
Member of first cohort								
Yes	495	344	1.43 (1.24–1.65)	1.29 (1.04–1.61)	0.0192			
No	330	296	1.1 (0.94–1.3)	Reference	–			
Baseline *Plasmodium* infection								
Positive	375	152	2.44 (2.10–2.85)	2.67 (2.17–3.3)	< 0.001			
Negative	450	487	0.91 (0.79–1.06)	Reference	–			
Inverse distance-weighted malaria prevalence								
Lowest	169	213	0.77 (0.62–0.97)	0.39 (0.30–0.52)	< 0.001	0.58 (0.38–0.89)	0.42 (0.31–0.57)	<0.001
Middle	241	216	1.11 (0.92–1.34)	0.57 (0.44–0.72)	< 0.001	0.84 (0.56–1.25)	0.61 (0.48–0.78)	<0.001
Highest	415	210	1.96 (1.68–2.29)	Reference	–	1.37 (0.96–1.97)	Reference	–
Malaria season								
Apr–June (high)	336	147	2.25 (1.99–2.55)	2.28 (1.97–2.65)	< 0.001	1.33 (0.93–1.92)	2.31 (1.98–2.7)	<0.001
July–Dec (low)	488	493	0.99 (0.86–1.13)	Reference	–	0.58 (0.4–0.83)	Reference	–
Number of ITNs < 300 m								
Lowest	315	208	1.50 (1.26–1.79)	1.99 (1.49–2.65)	< 0.001	1.02 (0.69–1.49)	1.48 (1.1–2)	0.010
Middle	345	217	1.58 (1.33–1.86)	2.08 (1.57–2.77)	< 0.001	0.97 (0.64–1.45)	1.41 (1.03–1.92)	0.031
Highest	165	215	0.76 (0.60–0.95)	Reference	–	0.69 (0.46–1.02)	Reference	–
Altitude								
Lowest	251	217	1.14 (0.95–1.36)	1.25 (0.96–1.64)	0.101	0.94 (0.65–1.38)	1.60 (1.21–2.13)	0.001
Middle	377	207	1.81 (1.52–2.14)	1.99 (1.52–2.59)	< 0.001	1.21 (0.82–1.8)	2.06 (1.57–2.69)	<0.001
Highest	197	216	0.91 (0.74–1.11)	Reference	–	0.59 (0.4–0.88)	Reference	–
Wealth index								
Poorest	285	212	1.34 (1.12–1.6)	1.46 (1.12–1.91)	0.006	0.89 (0.6–1.32)	1.26 (0.97–1.64)	0.085
Middle	334	210	1.57 (1.31–1.87)	1.71 (1.31–2.23)	< 0.001	1.07 (0.74–1.56)	1.52 (1.18–1.97)	0.001
Least poor	199	214	0.92 (0.75–1.12)	Reference	–	0.70 (0.47–1.05)	Reference	–
Caregiver completed primary school								
Yes	110	134	0.82 (0.60–1.11)	0.59 (0.42–0.81)	0.001	0.69 (0.44–1.07)	0.62 (0.45–0.85)	0.003
No	708	503	1.39 (1.24–1.56)	Reference	–	1.12 (0.79–1.57)	Reference	–

*ITN* insecticide-treated bed net, *iPPY* infections per person-year

Unadjusted malaria incidence did not differ by age or sex. Users of ITNs experienced similar incidence of malaria (1.27 iPPY) as non-ITN users (1.35 iPPY) (RR: 0.94, 95% CI 0.60–1.49, p = 0.801). Higher malaria incidence was significantly associated with being a member of the first cohort, higher baseline *Plasmodium* prevalence, higher IDW malaria prevalence, high malaria transmission season (April to June 2013), and fewer ITNs within a 300-m radius. Children living in wealthier households and those who had primary caregivers that completed primary school experienced significantly lower malaria incidence (Table 2).

After adjusting for age, IDW malaria prevalence, malaria season, number of ITNs within 300 m, household altitude, household wealth, and caregiver education, malaria incidence was slightly lower among children using ITNs compared to those who did not use ITNs (adjusted RR [aRR]: 0.83, 95% CI 0.54–1.27), but the PE of 16.7% was not statistically significant (p = 0.389) (Table 2).

### Effect of holes in ITNs on malaria incidence

Trends in malaria incidence by ITN use and use of ITNs with varying size holes were explored in Table 3. Malaria incidence was lowest among ITN users of nets with no holes (adjusted iPPY 0.75, 95% CI 0.55–1.02; Table 3) and there was no association with increasing size of holes in ITNs and malaria incidence.

**Table 3:** Malaria incidence by ITN use and net hole size in a fixed cohort of 1223 children, Liwonde, Malawi April–December 2013

ITN use and hole category	Malaria infections, n	PYs at risk	Adjusted iPPY (95% CI)
Use of ITN with no holes	393	345	0.75 (0.55–1.02)
Use of ITN with ≥ 1 small or medium hole (no large or extra-large holes)	208	122	0.98 (0.70–1.37)
Use of ITN with ≥ 1 large or extra-large hole	168	126	0.86 (0.61–1.21)
No ITN use	25	19	0.99 (0.59–1.66)

*ITN* insecticide-treated bed net, *iPPY* infections per person-year; *Large hole* between fist and head-sized, *Extra-large hole* head-sized or larger

* Adjusted for age, inverse distance-weighted malaria prevalence, malaria season, number of ITNs < 300 m, altitude, household wealth index, and caregiver completion of primary school

Given the lack of clear trend between size of holes in ITNs and malaria incidence, the effect of holes in ITNs was examined by considering use of ITNs with no holes and use of ITNs with ≥ 1 hole. Use of ITNs with no holes was recorded for 345 PYAR (58%). After adjusting for potential confounders, malaria incidence was significantly lower in children who used ITNs with no holes (of any size) compared to children who used ITNs with ≥ 1 hole (aRR: 0.82, 95% CI 0.69–0.98; p = 0.0268; Table 4), although the magnitude of the effect was similar to ITN users versus non-ITN users.

**Table 4:** Effect of net holes on malaria incidence in a fixed cohort of 1223 children, Liwonde, Malawi April–December 2013

ITN use and hole category	Malaria infections, n	PYs at risk	Multivariate*				
			Adjusted iPPY (95% CI)	Rate ratio (95% CI)	p-value	Rate ratio (95% CI)	p-value
Use of ITN with no holes	393	345	0.75 (0.55–1.02)	0.75 (0.49–1.17)	0.203	0.82 (0.69–0.98)	0.0268
Use of ITN with ≥ 1 hole of any size	376	251	0.91 (0.66–1.24)	0.91 (0.59–1.42)	0.694	Reference	–
No ITN use	25	19	0.99 (0.59–1.65)	Reference	–	–	–

*ITN* insecticide-treated bed net, *iPPY* infections per person-year

* Adjusted for age, inverse distance-weighted malaria prevalence, malaria season, number of ITNs < 300 m, altitude, household wealth index, and caregiver completion of primary school

## Discussion

In a setting of moderate pyrethroid resistance where mosquito mortality to pyrethroids ranged from 25 to 38% in *An. funestus* and 53–57% in *An. gambiae* s.l., malaria infection among users of ITNs that were 1–2 years old (some with holes) was lower than infection among non-users of ITNs, although the number of non-users was very small and the reduction was not statistically significant. Among users of ITNs, use of ITNs with no holes significantly reduced malaria incidence compared to use of ITNs with holes, with a similar magnitude of reduction as comparing the use of ITNs *versus* no ITNs. In contrast, the results from the first cohort in the same study area showed that new ITNs provided significant personal protection to users compared to non-users during the previous year (RR 0.7, 95% CI 0.5–0.8) [7]. Collectively, these findings suggest that, in a setting of moderate pyrethroid resistance and older ITNs, ITNs without holes may still provide partial protection, but the community effect and personal protection may be waning.

There could be several explanations for the lack of significant PE of ITNs observed in this study. First, given the high use of ITNs (> 95%) by participants, the study was underpowered to detect significant differences in malaria incidence between ITN users and non-users. Although the effect was imprecise, the direction suggested that ITN use was protective (PE: 17%). An effect of similar magnitude was found in the cohort enrolled at the western Kenya site of a large multi-country study, which also had high use of ITNs (> 90%, but lower than the present study) and high levels of pyrethroid resistance, but the results in the Kenya study were statistically significant [8, 9]. Second, the small reduction in malaria incidence between users and non-users of ITNs could be a result of reduced personal protection of ITNs, in the context of moderate levels of pyrethroid resistance and deterioration of the ITNs. Malaria incidence was lowest among users of ITNs with no holes and was similar among users of ITNs with holes and non-users of ITNs. The PE of using an ITN with no holes compared to no ITN use was higher than that for using an ITN with no holes versus an ITN with holes (PE 25% *versus* 18%); however, only the latter was statistically significant, likely because of limited power due to the low number of non-users of ITNs. Additionally, there was no clear trend in malaria incidence and use of ITNs with holes of increasingly larger size. The threshold at which a net is deemed “damaged” has been debated and may depend on the size, number and location of holes and the criteria by which (i.e. field based or laboratory-based) holes are characterized [34, 35]. Several studies have shown an association between nets with greater damage (i.e. larger holes or poorer condition) and increased malaria [15, 36], while other studies have indicated no association [37, 38]. None of these studies have examined the effect of ITNs with holes on malaria prospectively. The ITNs used by participants in this study were approximately 1 to 2 years old and a large proportion (35%) had holes of any size at the start of the second cohort study. Although ITN holes were assessed throughout the study and concurrently with ITN use based on caregiver self-report, the field-based categorization used may overestimate [35] the size of holes. These findings suggest that holes of any size may increase the risk of malaria infection among ITN users in a setting of moderate pyrethroid resistance. The loss of personal protection mirrors results from entomological studies which found that pyrethroid resistant mosquitoes were able to successfully feed on the occupants of nets as their physical condition deteriorated [10, 11].

Despite the absence of observed personal protection among users of ITNs with declining physical integrity, there was a significant association between a higher number of nets within a 300-m radius and lower malaria incidence, suggesting some evidence of a community effect [3]. There was a larger reduction in malaria incidence among ITN users compared to non-ITN users in the first cohort, which suggests that the community effect may have weakened. The apparent decline in personal protection but evidence of a community effect is somewhat contradictory. However, ITN use was high in the study area and despite the moderate levels of pyrethroid resistance, ITNs likely provided some protection, even if primarily as a physical barrier. Furthermore, measures of insecticide resistance are usually based on 24-hour survivorship after exposure to a fixed dose for a fixed time. Recent studies have suggested a delayed effect of insecticide exposure on mosquito mortality which may partially explain the apparent continued effectiveness of ITNs despite high levels of pyrethroid resistance throughout much of sub-Saharan Africa [39]. The presence of a community effect may also partially explain the lack of personal protection as non-users of ITNs receive some protection from malaria transmission, reducing the differences in incidence between users and non-users of ITNs [40].

Phenotypic and molecular evidence have demonstrated that pyrethroid resistance in *An. funestus* in Malawi is largely driven by oxidase enzymes that contribute to metabolic detoxification of pyrethroids [41, 42]. In settings of moderate intensity of monooxygenase-based resistance, such as Malawi, the WHO recommends the deployment of ITNs with the synergist piperonyl-butoxide (PBO) [43]. PBO inhibits certain mosquito oxidase enzymes, which prevents metabolization of the insecticide. A cluster-randomized trial in setting of high pyrethroid resistance in Tanzania demonstrated that malaria prevalence was significantly lower in clusters that received PBO ITNs compared to pyrethroid-only ITNs up to 21 months post-deployment [44]. Similarly, a mathematical modeling study showed that although new ITNs are highly protective even at higher levels or resistance, their effectiveness may rapidly wane as the net deterioration and declining insecticide content compromises the mass killing effect [45]. The study also found that deployment of PBO ITNs could avert up to 0.5 clinical malaria cases in settings of moderate pyrethroid resistance compared to pyrethroid-only ITNs [45]. Given the oxidase based resistance observed in *An. funestus* and the substantial impact of PBO ITNs on malaria in Tanzania, PBO ITNs are likely to provide additional benefit in Malawi. However, a recent study in an area of high oxidase-based pyrethroid resistance in Mozambique found complete loss of *An. funestus* susceptibility following exposure to permethrin or deltamethrin that was only moderately restored after pre-exposure to PBO [46] suggesting that other next-generation ITNs incorporating chlorfenapyr [47] or pyriproxyfen [48] might be needed in settings of high pyrethroid resistance. In Malawi, Centers for Disease Control and Prevention bottle bioassays indicated that *An. funestus* from two sites are susceptible to chlorfenapyr and that susceptibility to pyrethroids is restored after pre-exposure to PBO (T. Mzilahowa and J. Gimnig, unpublished data). However, bed net efficacy analyses conducted on mosquitoes from southern Malawi in 2014 indicated only partial restoration of susceptibility after exposure to the PBO Olyset^®^ Plus net (24-hour mortality rate of 67%). Thus, additional field studies might be needed to confirm the choice between PBO and next-generation ITNs in Malawi.

This study had several limitations. High ITN use reduced statistical power to detect differences in malaria incidence between ITN users and non-users. Although potential confounders were carefully ascertained and adjusted for in analyses, it is possible that residual and/or unmeasured confounding (due to heterogeneity in insecticide resistance, condition of ITNs, and insecticide content) could have biased the findings of this observational study. While several observational studies have found limited impact of pyrethroid resistance on the effectiveness of ITNs [5–9], studies comparing PBO [44] or dual-treated ITNs [48] to pyrethroid-only ITNs have demonstrated a clear benefit of these nets over pyrethroid-only ITNs in cluster-randomized designs, suggesting that some of the protection, such as through the community effect, may be underestimated in observational studies of the impact of resistance on ITNs. Additionally, the measurement of the physical integrity of ITNs was imperfect since caregiver self-report may be subject to recall and social desirability biases and assessing the presence or absence of holes of varying size does not capture other forms of net deterioration (e.g. many small holes compared to a single large hole) that can lead to increased malaria risk. Also, the insecticide content on the ITNs used by study participants was not measured, which could also provide an explanation for the minimal protection provided by ITNs in the present study. Measurement of insecticide content requires destructive sampling of nets followed by intensive laboratory analysis and results may be variable due to factors such as sun exposure, washing, and net storage. Finally, enrolled participants were significantly more likely to use ITNs compared to non-enrolled participants (84% vs 75%, p < 0.0001), raising the potential for selection bias if study participation was also associated with malaria risk. Because malaria testing was not performed during the second census, the association between study participation and malaria risk could not be evaluated directly.

Despite these limitations, this study provides evidence on the effectiveness of older ITNs in a setting where pyrethroid resistance has become a concern. These results are in line with other observational studies that indicate continued effectiveness of ITNs in areas with high pyrethroid resistance. However, recent cluster-randomized trials of PBO and next-generation ITNs suggest that observational studies may underestimate the impact of pyrethroid resistance. Furthermore, given that pyrethroid resistance in southern Africa is increasing, implementation of PBO or next-generation ITNs may help to maintain or increase the PE of ITNs in Malawi and similar settings, particularly as the physical integrity of ITNs deteriorates over time.

## Conclusions

In an area with moderate pyrethroid resistance and high use of older ITNs, the use of ITNs (some of which had holes) did not offer significant protection against incidence of malaria parasitaemia compared to no ITN use. However, among ITN users, ITNs without holes were significantly more protective against malaria compared to ITNs with holes. Additionally, an inverse relationship between number of ITNs in an area and malaria incidence was found, suggesting that high coverage of ITNs offered some community protection. These results suggest that ITNs without holes may continue to confer personal protection and ITNs (some with holes) may provide partial protection via a community effect in settings of moderate pyrethroid resistance. ITNs should remain an integral part of control programmes in malaria-endemic areas with pyrethroid resistance, and the deployment of next-generation ITNs such as PBO ITNs and dual-insecticide nets should be considered in these settings.

## Data Availability

The datasets generated and/or analysed during the current study are not publicly available but are available from the corresponding author on reasonable request.

## Abbreviations

CI: confidence interval
GEE: generalized estimating equations
HRP-2: histidine-rich protein 2
IDW: inverse distance-weighted
iPPY: incidence per person year
IQR: interquartile range
ITN: insecticide-treated bed net
Kdr: knockdown resistance
PBO: piperonyl-butoxide
PCR: polymerase chain reaction
PE: protective efficacy
PYAR: person-years at risk
RDT: rapid diagnostic test
RR: rate ratio
WHO: World Health Organization
